# Sex Assignment in Conditions Affecting Sex Development

**DOI:** 10.4274/jcrpe.2017.S009

**Published:** 2017-12-30

**Authors:** Renata Markosyan, S. Faisal Ahmed

**Affiliations:** 1 Yerevan State Medical University, Muratsan University Hospital, Clinic of Endocrinology, Yerevan, Armenia; 2 University of Glasgow School of Medicine, Developmental Endocrinology Research Group, Glasgow, United Kingdom

**Keywords:** Atypical, ambiguous, disorder of sex development, genitalia

## Abstract

The newborn infant with atypical genitalia presents a challenging clinical scenario and requires expert input. There have been appreciable advances in our knowledge of the underlying causes that may lead to a mere difference or a more serious disorder of sex development (DSD), the natural history of conditions, as well as the short and long-term complications of these conditions themselves, together with the clinical interventions that are associated with these conditions. With this information, the DSD expert can be more confident when discussing options with the parents of the newborn infant. By working within a multidisciplinary team, the expert should be able to support the family whilst individualising the management plan so that it is also cognizant of the shifts in societal attitudes and expectations around concepts of diversity and openness. It is, therefore, likely that the practice of assigning sex, especially in those cases where sex assignment is unclear on expert assessment, will continue to show temporal, social and geographical variations. It is imperative that clinical data for rare conditions such as these are collected in a standardized format and shared through a common registry so that any evidence that is used for future shifts in practice has a stronger foundation than that which is currently available.

## INTRODUCTION

When sex development is affected in early life, the involved infant often presents with atypical genita-lia in the neonatal period. This presentation raises the possibility of a disorder of sex development (DSD) (1). The underlying biological condition in a number of cases of atypical genitalia, especially those with a 46 XY karyotype who are raised as a boy, remains unclear. The newborn infant that has genitalia that are so atypical that a diagnosis cannot be reached at initial presentation, presents a problem of sex assignment and should be considered a clinical emergency. It is important to identify these scenarios as early as possible and to have a care pathway that can be quickly activated. The aim of this paper is to review the process of sex assignment and areas that are contentious and to consider future directions.

### Sex Development

Sex development is a process that can be broadly divided into the development of the gonads and the development of the reproductive organs and the genitalia. This process is under the control of molecular networks of male- and female-specific gene expression, dosing and interaction (2). Presence of XY chromosomes triggers activation of the SRY gene, which initiates development of a testis, where the primary sex cords develop into Sertoli cells. Sertoli cells produce anti-Müllerian Hormone (AMH) which promotes the regression of the Müllerian ducts. Leydig cells form outside the testicular tubules and produce testosterone, which stimulates the Wölffian duct to persist to form the epididymis, vas deferens and seminal vesicles. Under the influence of androgens, the genital tubercle differentiates and enlarges to become a penis, the urethral folds form the penile urethra, and labioscrotal swellings fuse to form the scrotum. In the absence of testicular development being switched on by the SRY gene on the Y chromosome, Wnt-4 signaling sustains oocyte and granulosa cell development, and suppresses Sertoli and Leydig cell differentiation. The Müllerian system of the embryo gives rise to the uterus, cervix, upper vagina, and fallopian tubes in the absence of AMH. In the absence of androgens, the phallus becomes a clitoris, the labioscrotal folds become the labia, and the urethra does not migrate to the tip of the phallus (2).

### Disorders of Sex Development

 “DSD” is an umbrella term for a group of conditions that arise due to a biological variation in chromosomal, gonadal, or anatomic sex. The current classification of DSD in three subgroups, sex chromosome DSD, 46, XX DSD, and 46,XY DSD, was recommended by the international consensus group on management of intersex disorders in Chicago in 2005 (1). These disorders could be determined at different development stages of the life-cycle in fetuses or newborns with atypical external genitalia, dysgenetic gonads and internal genitalia. The term ‘DSD’, by itself, is not a diagnosis but a presentation characterised by a wide range of clinical features such as hypospadias (1 in 250 boys), ambiguous genitalia (1 in 4500 live births) and complete XX or XY sex reversal (1 in 20,000 births) (3,4,5). Older children and adolescents may present with clinical features such as delayed puberty, unexpected virilization or gynaecomastia, infertility, or gonadal tumors.

The first step in sexual differentiation is the activation of the SRY gene to trigger testicular development at 7-8 weeks of fetal development. When there is a mutation or deletion of SRY, or one of the early downstream genes in gonadal differentiation, then the gonads fail to mature into either ovary or testis and become nonfunctional streak gonads. Failure of testicular development leads to absent male hormones required for masculinization of both internal and external genitalia. This leads to regression of the Wölffian duct and preservation of the Müllerian duct. The external genitalia continue on the female developmental pathway, leading to a normal external female phenotype at birth. Whilst the vagina and uterus form normally in the absence of AMH, the formation of functioning ovaries requires the activation of critical ovarian development genes.

In females with non-disjunction of the sex chromosomes, leading to the 45, X genotype (Turner syndrome), the primitive germ cells are displaced from the caudal yolk-sac into the indifferent gonad. Therefore absence of the second X chromosome leads to abnormal development of the follicles. This in turn leads to premature senescence in early childhood. The germ cells undergo premature death, sometime between late foetal life and the first few years after birth. Early biopsy of the gonad, at birth or shortly afterwards, may show some primary follicles which degrade over the next few years. As a result, for patients with 45, X/46, XX mosaicism ovarian function may be occasionally sustained until later in life. In some rare forms of abnormal sex determination, there is complete sex reversal, with XY females or XX males. In the latter case, the common cause is translocation of a small segment of the Y chromosome, which includes the SRY gene, onto the X chromosome, usually at Xp11 .3. Currently this is identified by fluorescent in situ hybridisation with a marker for the SRY gene (6).

### Factors That Influence Sex Assignment at Birth

The approach to sex of rearing decisions in DSD patients has changed fundamentally over time and involves many factors. Influencing factors for sex assignment include diagnosis, genital appearance, fertility potential, therapeutic and surgical options and familial views or circumstances including cultural biases. When a specific diagnosis can be reached, recommendations for sex assignment can be based upon outcome data. The assessment of the genitalia must include a description and symmetry of the external genital development including degree of virilization, Prader staging and the presence and position of gonads. Asymmetry is primarily seen as a result of greater virilization of the labioscrotal fold derived structures on one side compared with the other. This commonly results in the appearance of one side more like a labial fold and the other like a hemi-scrotum. For underdeveloped male genitalia, the capacity to respond to exogenous androgen may be a challenging method for determining sex assignment given that there are no agreed norms. Parental backgrounds and expectations, broader family dynamics, social circumstance and ethnic or cultural influences must also be considered in each case.

### Temporal Trends in Attitudes

The Chicago Consensus recommended that every affected child had the right to be assigned sex and generally sex assignment is performed soon after birth. However, most health care providers allow a period where notification of birth can be delayed. In some countries such as Australia, Bangladesh, Germany, India, New Zealand, Nepal and Pakistan, the sex of the child can be registered as undetermined and the calls for this category to be more widely available internationally as well as removing sex assignment from official documents is increasing. It is possible that the need for sex in official documents such as birth certificates or passports may have been driven by the need for sex to be a distinguishing marker of identification. With increasing availability of alternative forms of biometrics, the need to have sex as a marker of identification may reduce over time. In some infants affected by DSD and especially those presenting with genital ambiguity, the issue of sex of rearing has been a debatable aspect of management. In 2006, it was stipulated that sex assignment cannot solely be based on genital appearance but should include the diagnosis, surgical options, replacement therapy, the potential for fertility, views of the family and circumstances relating to cultural approach (3). The presentation of DSD in the newborn when sex assignment is unclear has often been considered “a medical and social emergency”. Whilst it is true that such a presentation may signify life threatening conditions such as congenital adrenal hyperplasia (CAH), a label of emergency may lead to a hastened process with inadequate communication within the team or with the family. More recently, in some countries such as Germany, parents have been given the option to delay sex assignment for longer than was previously possible and this may help with the process of sex assignment. It remains to be further studied whether these shifts in policy reduce the stigma or isolation felt by the parents or the child (7).

Recent data from the I-DSD Registry show that practice amongst specialist centres is also changing. Whereas in the past, infants with XY DSD (other than complete AIS) who had a very low external masculinization score were raised as a girl, more recently, these infants are more likely to be raised as a boy (8). Whilst this shift in practice is guided by accumulating evidence of adverse psychosocial and psychosexual outcome in those raised as girls (9), there is a continuing need to gather evidence on long-term outcome in those who are now being raised as boys. Whilst it is generally believed that 46 XX infants with CAH should be raised as girls, with the availability of long-term outcome data, some experts have questioned this practice in those infants who are severely virilised at birth, advocating that a male sex of rearing may be more appropriate (10).

### Sex Assignment

The birth of a child with suspected DSD is a challenging situation for parents and health professionals (11). In many cases, a decision is made immediately after birth about the sex of the child. The possible course of future physical, emotional and sexual development of individuals with DSD must also be kept in mind, in order to make the right decision in childhood to achieve good lifelong outcomes for health, emotional and social development (12). The lack of knowledge about the relative contribution of biological (e.g., genes and prenatal sex hormone exposure) and non-biological influences (e.g., parental attitude, peer influences and cultural context) on gender development can make sex assignment more difficult. Prediction of adult gender identity is difficult in some conditions. Although there is no doubt that investigations are required in all infants with suspected DSD, there is less certainty about when investigations should be performed in those cases in which the genitalia are less ambiguous. Expert opinion suggests that groups of infants who should be evaluated include those with female genitalia with atypical features, such as an enlarged clitoris, or those with male genitalia with atypical features (13,14). Also, evaluation may be necessary in those who have a family history of DSD or there is discordance between genital appearance and a prenatal karyotype. The health care team has the important role of evaluating the patient and informing the parents about the diagnosis, possible therapies, available outcome data as well as availability of support groups (12). Surgical possibilities, potential for fertility and the need for hormone replacement should also be taken into account when necessary.

### Geographical Differences

Society often plays a major role in the decision for sex assignment and the sex of rearing decision is often considered to be the parents’ right, obligation and responsibility. Strong social pressures influenced by cultural, traditional and economic factors persist in some social groups, where the male may have a dominant role in financial and social life. In such communities where a man is the traditional breadwinner, choosing the male gender is often considered to be more preferable for the affected offspring than the individual’s sexual potential (15,16,17). There are only a few reports about geographical differences in choosing sex of rearing. A recent study from India showed that seven infants who were 46, XX and had CAH were raised as males because of family preference, older age of diagnosis and having a “good” phallus (18). In such scenarios, the algorithm for sex assignment is over simplified and based on good or poor phallic development (19,20).

### Evidence of Discontent with Assigned Sex

It is not unusual that adults with DSD experience discontent with the assigned sex. This may be attributed to several reasons including medical interventions such as surgery or hormone replacement therapy, impact of delayed or precocious development, experience of stigmatization or psychological trauma, social expectation of gender role behavior and other coexisting mental health conditions. Some studies found that girls with CAH show masculinization of behavior, such as spatial orientation, visualization, targeting, personality, cognitive abilities, and sexuality (21,22,23). Others demonstrated a masculine bias on various personality traits supporting the determining role of parental steroids in sex-role identity (e.g., Detachment and Indirect Aggression Scales, Aggression and Stress Reaction Scales, Reinish’s Aggression Inventory) (24). Although women with CAH develop a female gender identity, gender dysphoria may be more common than in women without CAH (25). It was shown that five percent of adolescent and adult women with CAH suffer a form of gender dysphoria contributing to the decision for sex re-assignment. The extent of sexual activity of women with CAH may also be lower when compared with the normal population (26). A recent literature review concluded that people who were 46 XX and extremely virilized due to CAH and who were reared male may enjoy satisfactory level of social and sexual function as male adults if they obtained optimal social support (27,28). Prenatal androgen stimulation in girls with CAH results in different levels of virilization. The severity of the enzyme defect has influence on phenotype. Sexual function and the quality of sexual life in women with CAH following genital surgery with clitoroplasty and vaginoplasty has been reported in several small group studies and many report dissatisfaction with clitoral surgery (29,30,31). Medically, the low birth rate in women with CAH may be due to the influence of low gonadotropins and high progesterone levels (32).

Due to an androgen biosynthesis problem, children with 46,XY who have 5a reductase-type 2 deficiency or 17b-hydroxysteroid dehydrogenase-type 3 deficiency are usually born with female-appearing or ambiguous genitalia. In general, these infants are raised as girls and at puberty, when they start to masculinize, transition to the male role has been described (33). An increased rate of sex change from the female to the male sex role has been seen in children and adolescents with genital malformation (agenesis of the penis, cloacal exstrophy) who grow up as girls and had a normal level of male hormones at birth (34). The increase in testosterone level after puberty thus seems to be an important factor in gender identity and consolidation in individuals with these conditions. It is also possible that testosterone exposure at critical prenatal stages may have also played a role. Cultural factors should be considered, because gender role change may also occur at different rates in different societies (1).

In Complete Androgen Insensitivity syndrome (CAIS) the complete female appearance at birth usually masks the condition completely and the infants are raised without any doubt as girls. These children display typical girl behavior and female gender development, with no signs of gender dysphoria (27,34). However, it is possible that women with CAIS may be dissatisfied with their primary sex organs, even without observable gender atypical signs (35). The issue of insecurity based on their own body perception may arise due to discrepancy between gender role and karyotype. On the other hand, individuals with Partial Androgen Insensitivity syndrome (PAIS) may develop gender dysphoria (36). Approximately 25% of individuals with PAIS appear to develop gender dysphoria regardless of the sex they are reared as (37).

Many affected children with DSD undergo feminizing or masculinizing genitoplasties as well as gonadectomies. There are several reasons for these surgeries including aligning a child’s phenotype more closely with their sex of rearing, determining future fertility potential, and removing the risk of malignancy (38). In those undergoing feminizing surgeries (clitoroplasty and vaginoplasty) the total excision of the clitoris is no longer recommended. The current approach is a clitoroplasty that preserves the glans and neurovascular bundle of the phallus for better genital sensation and orgasmic potential (39). The point of entry of the vagina into the urogenital sinus is important for the choice of vaginoplasty procedure. Novel methods for vaginoplasty include skin flap, sigmoid bowel, and pullthrough (36). Alternative interventions such as vaginal dilatation may also be preferred in some situations. The timing and the need for these procedures is increasingly debatable and is beyond the scope of this review on sex assignment.

Recent investigations of outcomes of gonadectomy and vaginoplasty in girls and women affected by CAIS range from satisfaction with surgery (40,41) to preference for early surgery, to a lack of sexual desire/arousal and dyspareunia attributed to these procedures (42,43). Among the factors contributing to the high dissatisfaction with treatment in this subgroup are the lack of information provided to the patient about their condition and its management so that they can make an informed decision for themselves. It is unclear if improved surgical techniques have resulted in higher patient satisfaction, since age did not influence the satisfaction rates with surgery (42). On the other hand, women with 46,XY DSD without genitoplasty and born with female external genitalia were mostly satisfied with their vaginal length and clitoral arousal (44). However, a recent Dutch study with a mix of people with XY DSD and CAH reported impairment on the female sexual function index and were at risk of developing sexual dysfunction, non-operated patients with CAIS and complete gonadal dysgenesis were signifi-cantly more dissatisfied with sexual life than operated women with XY DSD or CAH. This study showed that a large proportion of women reported problems of coping with diagnosis, distress of infertility and suffering from societal ignorance (43,44). It is therefore possible that these are the major contributory factors in the impairment of psychosexual and psychosocial life in XY DSD.

Masculinizing surgeries for DSD include release of ventral chordee, hypospadias repair, gonadectomies and placement of prosthetic testes in the scrotum at puberty. Many studies have found that men with hypospadias repair in childhood still report at least some degree of dissatisfaction with their genital appearance and size, which may lead to psychosexual distress and jeopardize sexual well-being (45). In 46 XY DSD with micropenis, it is not only the genital appearance, but also overall physical development - such as male development and eventual breast growth - as is the case in PAIS - that can lead to a negative body image and impaired social interactions (46). Retrospectively, it is increasingly clear that masculinizing genitoplasty in severe cases of hypospadias may require many more procedures than feminizing genitoplasty and may also result in a poorer cosmetic outcome (46). In comparison to those who develop a male gender, patients with 46,XY DSD reared male who ultimately develop a female gender do not experience different cosmetic or functional outcomes from their genitoplasty (44). Postoperative complications (fistulae, urethral strictures and meatal stenosis and repeated surgical procedures are of particular prospective concern because of associated scarring and loss of tissue, as well as the estimated negative impact on sexual function (47). In addition, penile lengthening procedures, such as in hypospadias repair in men with penile deficiency, can only elongate the penis by an average of 1.5-2.5 cm. Whether or not a correlation exists between small penile length and dysfunctional penetrative intercourse remain unclear, although a penile length of more than 6-7 cm seems to constitute a premise for successful sexual contact (48,49,50). Some authors described a few men with micropenis who reported a mutually satisfying sex life with their heterosexual partners (51,52).

### Summary

Differences in DSD management will result from a combination of traditional beliefs, folk remedies and prejudices, fed by rumour and discrimination and available healthcare resources and expertise (18). Nevertheless, it should be appreciated that people with DSD have the same desires as everyone else: to find a peer who will love them; to be a valuable part of society; to be comfortable with their body; to be able to have satisfactory sexual relations; to integrate into the community; and to trust their medical caregivers. More clinical studies as well as academic and public debate are needed to support people with DSD with sex assignment, gender identity development, atypical gender role behavior, sexual orientation and satisfaction with their own sexuality. It is debatable whether the dissatisfaction that people with DSD experience with the allocated sex of rearing is a gender identity disorder or not. People with DSD who are discontent may simply be showing an evolving discrepancy between the gender identity they experience and the sex of rearing which, in most cases was chosen by their carers at birth. All carers, parents and professionals, should be aware that possibility of dissatisfaction with the assigned sex, however small, does exist and centres that provide expert care should be prepared to support the patient and the family, if required.

Finally, healthcare workers should share expertise and collaborate globally in prospective studies as it is essential to gain insight into the outcome of individuals affected by these rare conditions. The variations in practice can be decreased through networks of clinical and research centers. Disease registries are playing a significant role in development and improvement of networks. Establishment of the DSD registry in 2007, initially as the ESPE DSD Registry, followed by the Euro-DSD Registry and currently as the I-DSD Registry, is a perfect example of how registries can evolve and also be used to address issues ranging from fundamental mechanisms to clinical practice and health care outcomes (8). It is likely that newly established international collaborations to generate sufficient numbers for the study of very rare disorders will provide better information on which new protocols can be developed.
